# Designing Ising machines with higher order spin interactions and their application in solving combinatorial optimization

**DOI:** 10.1038/s41598-023-36531-4

**Published:** 2023-06-12

**Authors:** Mohammad Khairul Bashar, Nikhil Shukla

**Affiliations:** Department of Electrical and Computer Engineering, University of Virginia, Charlottesville, VA 22904 USA

**Keywords:** Mathematics and computing, Applied physics, Statistical physics, thermodynamics and nonlinear dynamics, Electrical and electronic engineering

## Abstract

The Ising model provides a natural mapping for many computationally hard combinatorial optimization problems (COPs). Consequently, dynamical system-inspired computing models and hardware platforms that minimize the Ising Hamiltonian, have recently been proposed as a potential candidate for solving COPs, with the promise of significant performance benefit. However, prior work on designing dynamical systems as Ising machines has primarily considered quadratic interactions among the nodes. Dynamical systems and models considering higher order interactions among the Ising spins remain largely unexplored, particularly for applications in computing. Therefore, in this work, we propose Ising spin-based dynamical systems that consider higher order (> 2) interactions among the Ising spins, which subsequently, enables us to develop computational models to *directly* solve many COPs that entail such higher order interactions (i.e., COPs on hypergraphs). Specifically, we demonstrate our approach by developing dynamical systems to compute the solution for the Boolean NAE-K-SAT (K ≥ 4) problem as well as solve the Max-K-Cut of a hypergraph. Our work advances the potential of the physics-inspired ‘toolbox’ for solving COPs.

## Introduction

The minimization of the Ising Hamiltonian using dynamical systems such as coupled electronic^1–5^ and photonic oscillators^6–8^ has received substantial attention in recent years^9,10^. A significant driving force behind the effort to realize a so-called ‘Ising machine’ is that the solution to the Ising model can be mapped to many computationally intractable problems in combinatorial optimization (e.g., MaxCut, Traveling Salesman Problem (TSP) among others)^11–18^. Consequently, this creates the possibility of realizing Ising machine-inspired custom accelerators that can offer the possibility of significant performance benefits. However, dynamical system formulations that have been used to ‘solve’ the Ising model typically consider only pair-wise coupling; examples include, oscillator Ising machines, coherent Ising machines etc. From an application standpoint, while these characteristics capture quadratic interactions, the dynamical systems and their supporting computational models cannot be applied *directly* to solve problems that require higher order interaction among the spins^19,20^. Therefore, the objective of this work is two-fold: (1) define dynamical systems that model higher order (> 2) interactions among the Ising spins; and (2) map the resulting dynamics to relevant computational problems. We consider two examples: computing the solutions for the NAE-K-SAT (Not-All-Equal SAT) problem and the Max-K-Cut of a hypergraph. Our motivation behind selecting these two combinatorial optimization problems was that their objective functions directly map to the solution of the higher order Ising models, and therefore, help illustrate the principle of how dynamical systems for the higher order Ising models can be used in combinatorial optimization. Also, we emphasize here that presently our focus is on defining the Ising machine dynamics that capture the higher order interactions, and not on the physical implementation of the higher order interactions.

The general form to represent higher order interactions among the Ising spins can be expressed as,

1 $$H=-{\sum }_{i,j}{J}_{ij}^{\left(2\right)}{s}_{i}{s}_{j}-{\sum }_{i,j,k}{J}_{ijk}^{\left(3\right)}{s}_{i}{s}_{j}{s}_{k}-{\sum }_{i,j,k,l}{J}_{ijkl}^{\left(4\right)}{s}_{i}{s}_{j}{s}_{k}{s}_{l}\dots .$$

where ${J}_{ij}^{\left(2\right)}$ represents the pairwise interaction coefficient between two Ising spins. The first term on the right-hand side ($-{\sum }_{i,j}{J}_{ij}^{\left(2\right)}{s}_{i}{s}_{j}$) is usually considered when describing quadratic/pairwise interactions among Ising spins $s={\left\{-\mathrm{1,1}\right\}}^{n}$; the Zeeman term which considers the interaction of spins with an external magnetic field has been neglected here. Considering the higher order interactions among the spins can help describe the objective functions of several combinatorial optimization problems (COPs) as illustrated here with the example of the NAE-K-SAT problem (without the need for problem decomposition). The NAE-K-SAT problem is a constrained version of the Boolean Satisfiability (SAT) problem where the objective is to find an assignment for the variables of the given Boolean expression (in the conjunctive normal form) such that: (a) at least one variable in every clause is TRUE (i.e., the clause is satisfied; standard SAT constraint); (b) at least one variable in every clause is FALSE^21^; the NAE-K-SAT problem is considered here since it directly maps to the general form of Eq. (1), as illustrated further on. Using an approach inspired by SAT, the NAE-K-SAT problem can be expressed as computing an assignment for the variables such that $Y \left(={C}_{1}.{S}_{1}\wedge {C}_{2}.{S}_{2}\wedge \dots \wedge {C}_{M}.{S}_{M}\right)=1.$ Here, ${C}_{i}\equiv ({x}_{1}\vee {x}_{2}\vee {\overline{x} }_{3}\dots {\overline{x} }_{N})$, and ${S}_{i}\equiv ({\overline{x} }_{1}\vee {\overline{x} }_{2}\vee {x}_{3}\dots {x}_{N})$ (i.e., ${S}_{i}$ and ${C}_{i}$ have the same variables but in opposite forms). Traditionally, when considering only pairwise interactions among the Ising spins, mapping such problems can entail significant pre-processing including the use of auxiliary variables that can significantly increase the size of the problem that must be eventually solved^20,22–25^ using the dynamical system.

## Results

### NAE-4-SAT

To illustrate how we can map the NAE-K-SAT problem to higher order interactions among the Ising spins, we first consider the example of the NAE-4-SAT problem where each clause of the NAE-4-SAT problem consists of 4 literals, expressed in the general form as $\left({x}_{i}\vee {x}_{j}\vee {x}_{k}\vee {x}_{l}\right).\left({\overline{x} }_{i}\vee {\overline{x} }_{j}\vee {\overline{x} }_{k}\vee {\overline{x} }_{l}\right)\equiv \left({x}_{i}\oplus {x}_{j}\right)\vee \left({x}_{i}\oplus {x}_{k}\right)\vee \left({x}_{i}\oplus {x}_{l}\right)\vee \left({x}_{j}\oplus {x}_{k}\right)\vee \left({x}_{j}\oplus {x}_{l}\right)\vee \left({x}_{k}\oplus {x}_{l}\right)$, where $x\in {\{\mathrm{0,1}\}}^{n}$ ($x$ is a set of Boolean variables). K = 4 is specifically chosen here since it is the lowest K where higher order interactions among the Ising spins are required to formulate the objective function for the problem (shown in Table 1). To formulate the problem in terms of Ising spins, we utilize the following property among the Boolean variables and the spins $\left({x}_{i}\oplus {x}_{j}\right)\equiv \frac{1-{s}_{i}{s}_{j}}{2}$. Here, the logic level 0 (1) corresponds to an evaluation of − 1(1) of the expression on the right-hand side, respectively. Furthermore, the complement of the logical OR among the XOR terms ($\left({x}_{i}\oplus {x}_{j}\right)\vee \left({x}_{i}\oplus {x}_{k}\right)\vee \dots \vee \left({x}_{k}\oplus {x}_{l}\right)$) can be expressed as, $\left(1-\left(\frac{1-{s}_{i}{s}_{j}}{2}\right)\right).\left(1-\left(\frac{1-{s}_{i}{s}_{k}}{2}\right)\right)\dots \left(1-\left(\frac{1-{s}_{k}{s}_{l}}{2}\right)\right)$. Simplifying the above expression yields $\left(\frac{1+{s}_{i}{s}_{j}}{2}\right)\left(\frac{1+{s}_{i}{s}_{k}}{2}\right)\left(\frac{1+{s}_{i}{s}_{l}}{2}\right)\dots \left(\frac{1+{s}_{k}{s}_{l}}{2}\right)\equiv \frac{1}{8}(1+{s}_{i}{s}_{j}+{s}_{i}{s}_{k}+{s}_{i}{s}_{l}+{s}_{j}{s}_{k}+{s}_{j}{s}_{l}+{s}_{k}{s}_{l}+{s}_{i}{s}_{j}{s}_{k}{s}_{l})$. It can be observed that besides the second order interaction terms, the resulting expression also contains a 4^th^ order interaction term among the spins. Consequently, the objective function for the NAE-4-SAT problem, over M clauses, can be formulated as the minimization of

**Table 1:** Objective functions for the NAE-K-SAT problem expressed using Ising spins.

K	Expression for a single clause & objective function for the NAE-K-SAT
2	Expression for a single clause: $\left({x}_{i}\vee {x}_{j}\right).\left({\overline{x} }_{i}\vee {\overline{x} }_{j}\right)\equiv {s}_{i}{s}_{j}$
	Objective function: $H=-\sum_{m=1}^{M}\sum_{i,j,i<j}^{N}\left(-{c}_{mi}{c}_{mj}{s}_{i}{s}_{j}\right)\equiv -\sum_{m=1}^{M}\sum_{i,j,i<j}^{N}{J}_{ij}{s}_{i}{s}_{j}$ Where ${\mathrm{J}}_{\mathrm{ij}}=-{\mathrm{c}}_{\mathrm{mi}}{\mathrm{c}}_{\mathrm{mj}}.$ It can be observed that when the variables appear only in the normal form i.e., ${\mathrm{c}}_{\mathrm{mi}}\ge 0$, the expression represents the solution to the archetypal MaxCut problem
3	Expression for a single clause: $\left({x}_{i}\vee {x}_{j}\vee {x}_{k}\right).\left({\overline{x} }_{i}\vee {\overline{x} }_{j}\vee {\overline{x} }_{k}\right)\equiv {s}_{i}{s}_{j}+{s}_{i}{s}_{k}+{s}_{j}{s}_{k}$
	Objective function: $H=-\sum_{m=1}^{M}\sum_{i,j,i<j}^{N}\left({-c}_{mi}{c}_{mj}{s}_{i}{s}_{j}\right)$
4	Expression for a single clause: $\left({x}_{i}\vee {x}_{j}\vee {x}_{k}\vee {x}_{l}\right).\left({\overline{x} }_{i}\vee {\overline{x} }_{j}\vee {\overline{x} }_{k}{\vee \overline{x} }_{l}\right)\equiv {s}_{i}{s}_{j}+{s}_{i}{s}_{k}+{s}_{i}{s}_{l}+{s}_{j}{s}_{k}+{s}_{j}{s}_{l}+{s}_{k}{s}_{l}+{s}_{i}{s}_{j}{s}_{k}{s}_{l}$
	Objective function: $$H=-{\sum }_{m=1}^{M}\left(\sum_{\begin{array}{c}i,j\\ i<j\end{array}}^{N}\left({-c}_{mi}{c}_{mj}{s}_{i}{s}_{j}\right)+\sum_{\begin{array}{c}i,j,k,l\\ i<j<k<l\end{array}}^{N}\left(-{c}_{mi}{c}_{mj}{c}_{mk}{c}_{ml}{s}_{i}{s}_{j}{s}_{k}{s}_{l}\right)\right)$$
5	Expression for a single clause: $\left({x}_{i}\vee {x}_{j}\vee {x}_{k}\vee {x}_{l}\vee {x}_{m}\right).\left({\overline{x} }_{i}\vee {\overline{x} }_{j}\vee {\overline{x} }_{k}{\vee \overline{x} }_{l}{\vee \overline{x} }_{m}\right)$ $\equiv {s}_{i}{s}_{j}+{s}_{i}{s}_{k}+{s}_{i}{s}_{l}+{s}_{i}{s}_{m}+{s}_{j}{s}_{k}+{s}_{j}{s}_{l}+{s}_{j}{s}_{m}+{s}_{k}{s}_{l}+{s}_{k}{s}_{m}+{s}_{l}{s}_{m}+{s}_{i}{s}_{j}{s}_{k}{s}_{l}+{s}_{i}{s}_{j}{s}_{k}{s}_{m}+{s}_{i}{s}_{j}{s}_{l}{s}_{m}+{s}_{i}{s}_{k}{s}_{l}{s}_{m}+{s}_{j}{s}_{k}{s}_{l}{s}_{m}$
	Objective function: $$H=-{\sum }_{m=1}^{M}\left(\sum_{\begin{array}{c}i,j\\ i<j\end{array}}^{N}\left(-{c}_{mi}{c}_{mj}{s}_{i}{s}_{j}\right)+\sum_{\begin{array}{c}i,j,k,l\\ i<j<k<l\end{array}}^{N}\left(-{c}_{mi}{c}_{mj}{c}_{mk}{c}_{ml}{s}_{i}{s}_{j}{s}_{k}{s}_{l}\right)\right)$$

We note that constants and scalars have not been shown here in the expression for the single clause as well as for the objective function.

2 $${H}_{NAE-4-SAT}=-{\sum }_{m=1}^{M}\left(\sum_{\begin{array}{c}i,j\\ i<j\end{array}}^{N}\left({-c}_{mi}{c}_{mj}{s}_{i}{s}_{j}\right)+\sum_{\begin{array}{c}i,j,k,l\\ i<j<k<l\end{array}}^{N}\left({-c}_{mi}{c}_{mj}{c}_{mk}{c}_{ml}{s}_{i}{s}_{j}{s}_{k}{s}_{l}\right)\right)$$

Here, ${c}_{mi}=1(-1)$, if the *i*th variable appears in the $m\mathrm{th}$ clause in the normal (negated) form; ${c}_{mi}=0$ if the *i*$\mathrm{th}$ variable is absent from the $m\mathrm{th}$ clause. Using the same approach, we derive such expressions for a few other values of K in the NAE-K-SAT problem in Table 1. Details of the derivation of the objective function for NAE-5-SAT are shown in Supplementary 1.

#### Constructing a dynamical system for the NAE-K-SAT problem

We now aim to formulate the dynamical system and the corresponding energy function for the NAE-K-SAT problem. The dynamical system, defined by $-{\left({\nabla }_{\phi }E\right)}_{i}=\frac{d{\phi }_{i}}{dt}$, is designed such that the ground state of the ‘energy’ function (more precisely, the Lyapunov function) must correspond to a global optimum of the objective function. To construct this system, we draw inspiration from the dynamics of coupled oscillators under second harmonic injection which effectively forces the oscillator states to assume a binary phase value of 0 or π (details of the second harmonic injection can be found in work by Wang et al.^26^). Without loss of generality, we assume that one spin state (say, $s=+1$) is represented by phase 0 while the other spin state ($s=-1$) is represented by the phase angle π. Subsequently, the second order interaction terms among the Ising spins ${s}_{i}{s}_{j}$ can be represented by $\mathrm{cos}({\upphi }_{i}-{\upphi }_{j})$. When the spins are in opposite states i.e., ${s}_{i}=1\left(-1\right);{s}_{j}=-1\left(1\right)$, ${s}_{j}{s}_{j}\equiv \mathrm{cos}\left({\upphi }_{i}-{\upphi }_{j}\right)=-1$, whereas when the spins are in the same states i.e., ${s}_{i}=1\left(-1\right);{s}_{j}=1\left(-1\right)$, ${s}_{j}{s}_{j}\equiv \mathrm{cos}\left({\upphi }_{i}-{\upphi }_{j}\right)=1$. Similarly, the higher order interactions can be modeled as shown in Table 2.

**Table 2:** Equivalent energy function for modeling higher order interactions among Ising spins. The second harmonic signal included as a part of the dynamics (not shown here) helps force $\upphi$ to $\{0,\uppi \}$.

Order	Ising interaction	Equivalent formulation for constructing dynamical system
2	${s}_{i}{s}_{j}$	$\mathrm{cos}\left({\phi }_{i}-{\phi }_{j}\right)$
3	${s}_{i}{s}_{j}{s}_{k}$	$\mathrm{cos}\left({\phi }_{i}-{\phi }_{j}+{\phi }_{k}\right)$
4	${s}_{i}{s}_{j}{s}_{k}{s}_{l}$	$\mathrm{cos}\left({\phi }_{i}-{\phi }_{j}+{\phi }_{k}-{\phi }_{l}\right)$
5	${s}_{i}{s}_{j}{s}_{k}{s}_{l}{s}_{m}$	$\mathrm{cos}\left({\phi }_{i}-{\phi }_{j}+{\phi }_{k}-{\phi }_{l}+{\phi }_{m}\right)$
6	${s}_{i}{s}_{j}{s}_{k}{s}_{l}{s}_{m}{s}_{n}$	$\mathrm{cos}\left({\phi }_{i}-{\phi }_{j}+{\phi }_{k}-{\phi }_{l}+{\phi }_{m}-{\phi }_{n}\right)$

The equivalence between the higher order terms and the corresponding energy term is shown in Table 3.

**Table 3:** Equivalence between the higher order Ising spin interaction terms and the equivalent energy function.

Second order interactions (${s}_{i}.{s}_{j}$)			
${s}_{i}{ s}_{j}$	${s}_{i}.{s}_{j}$	${\phi }_{i} {\phi }_{j}$	$\mathrm{cos}\left({\phi }_{i}-{\phi }_{j}\right)$
− 1 − 1	+ 1	$\pi \pi$	+ 1
− 1 + 1	− 1	$\pi 0$	− 1
+ 1 − 1	− 1	$0 \pi$	− 1
+ 1 + 1	+ 1	$0 0$	+ 1

Third order interactions (${s}_{i}.{s}_{j}.{s}_{k}$)			
${s}_{i} {s}_{j} {s}_{k}$	${s}_{i}.{s}_{j}.{s}_{k}$	${\phi }_{i} {\phi }_{j}{ \phi }_{k}$	$\mathrm{cos}\left({\phi }_{i}-{\phi }_{j}+{\phi }_{k}\right)$
− 1 − 1 − 1	− 1	$\pi \pi \pi$	− 1
− 1 − 1 + 1	+ 1	$\pi \pi 0$	+ 1
− 1 + 1 − 1	+ 1	$\pi 0 \pi$	+ 1
− 1 + 1 + 1	− 1	$\pi 0 0$	− 1
+ 1 − 1 − 1	+ 1	$0 \pi \pi$	+ 1
+ 1 − 1 + 1	− 1	$0 \pi 0$	− 1
+ 1 + 1 − 1	− 1	$0 0 \pi$	− 1
+ 1 + 1 + 1	+ 1	$0 0 0$	+ 1

Fourth order interactions (${s}_{i}.{s}_{j}.{s}_{k}.{s}_{l}$)			
${s}_{i} {s}_{j} {s}_{k} {s}_{l}$	${s}_{i}.{s}_{j}.{s}_{k}.{s}_{l}$	${\phi }_{i} {\phi }_{j}{ \phi }_{k} {\phi }_{l}$	$\mathrm{cos}\left({\phi }_{i}-{\phi }_{j}+{\phi }_{k}-{\phi }_{l}\right)$
− 1 − 1 − 1 − 1	+ 1	$\pi \pi \pi \pi$	+ 1
− 1 − 1 − 1 + 1	− 1	$\pi \pi \pi 0$	− 1
− 1 − 1 + 1 − 1	− 1	$\pi \pi 0 \pi$	− 1
− 1 − 1 + 1 + 1	+ 1	$\pi \pi 0 0$	+ 1
− 1 + 1 − 1 − 1	− 1	$\pi 0 \pi \pi$	− 1
− 1 + 1 − 1 + 1	+ 1	$\pi 0 \pi 0$	+ 1
− 1 + 1 + 1 − 1	+ 1	$\pi 0 0 \pi$	+ 1
− 1 + 1 + 1 + 1	− 1	$\pi 0 0 0$	− 1
+ 1 − 1 − 1 − 1	− 1	$0 \pi \pi \pi$	− 1
+ 1 − 1 − 1 + 1	+ 1	$0 \pi \pi 0$	+ 1
+ 1 − 1 + 1 − 1	+ 1	$0 \pi 0 \pi$	+ 1
+ 1 − 1 + 1 + 1	− 1	$0 \pi 0 0$	− 1
+ 1 + 1 − 1 − 1	+ 1	$0 0 \pi \pi$	+ 1
+ 1 + 1 − 1 + 1	− 1	$0 0 \pi 0$	− 1
+ 1 + 1 + 1 − 1	− 1	$0 0 0 \pi$	− 1
+ 1 + 1 + 1 + 1	+ 1	$0 0 0 0$	+ 1

Using the above relationships developed in Table 1, the energy functions for the NAE-K-SAT problem can be formulated as shown in Table 4. The corresponding dynamics $\left(\frac{d{\phi }_{i}}{dt}\right)$, shown in Table 4, can be obtained from the dynamical system equation $\frac{d{\phi }_{i}}{dt}=-{\left({\nabla }_{\phi }E\right)}_{i}$. The second harmonic term in the energy function $\left(-\frac{{C}_{s}}{2}\sum_{i=1}^{N}\mathrm{cos}\left(2{\phi }_{i}\right)\right)$ is added to ensure that the oscillator phases effectively binarize to $\left\{0, \pi \right\}$. The energy contribution of this term is minimized ($=-N\frac{{C}_{s}}{2}$) at the binary phase points $\phi \in \left\{0, \pi \right\}$. Consequently, by using the appropriate strength of the second harmonic injection (${C}_{s}$), we can ensure that the energy function reaches its minimum for $\phi \in \left\{0, \pi \right\}$. We have borrowed this approach from prior work on oscillator-based Ising machines (with second order interactions)^26^.

**Table 4:** Objective functions, corresponding energy expressions, and system dynamics for NAE-K-SAT problems for K = 2, 3, 4, and 5. We note that while the form of the expressions for K = 2 and K = 3, as well as K = 4 and K = 5 are similar, the coefficients (${\mathrm{c}}_{\mathrm{mi}}$) are different. C is the strength of coupling among the nodes whereas ${\mathrm{C}}_{\mathrm{s}}$ represents the strength of the second harmonic injection.

K	Objective function, equivalent energy function, and dynamics
2 and 3	Objective function: $H=-\sum_{m=1}^{M}\sum_{i,j,i<j}^{N}\left(-{c}_{mi}{c}_{mj}{s}_{i}{s}_{j}\right)$
	Energy function: $E=C\sum_{m=1}^{M}\left[\sum_{i,j,i<j}^{N}{c}_{mi}{c}_{mj}\mathrm{cos}\left({\phi }_{i}-{\phi }_{j}\right)+1\right]-\frac{{C}_{s}}{2}\sum_{i=1}^{N}\mathrm{cos}\left(2{\phi }_{i}\right)$
	Dynamics: $\frac{d{\phi }_{i}}{dt}=C\left[\sum_{m=1}^{M}\sum_{j=1}^{N}{c}_{mi}{c}_{mj}\mathrm{sin}\left({\phi }_{i}-{\phi }_{j}\right)\right]-{C}_{s}\mathrm{sin}\left(2{\phi }_{i}\right)$
4 and 5	Objective function: $$H=-{\sum }_{m=1}^{M}\left(\sum_{\begin{array}{c}i,j\\ i<j\end{array}}^{N}\left({-c}_{mi}{c}_{mj}{s}_{i}{s}_{j}\right)+\sum_{\begin{array}{c}i,j,k,l\\ i<j<k<l\end{array}}^{N}\left({-c}_{mi}{c}_{mj}{c}_{mk}{c}_{ml}{s}_{i}{s}_{j}{s}_{k}{s}_{l}\right)\right)$$
	Energy function: $$E=C\sum_{m=1}^{M}\left[\sum_{i,j,i<j}^{N}{c}_{mi}{c}_{mj}\mathrm{cos}\left({\phi }_{i}-{\phi }_{j}\right)+\sum_{\begin{array}{c}i,j,k,l\\ i<j<k<l\end{array}}^{N}{c}_{mi}{c}_{mj}{c}_{mk}{c}_{ml}\mathrm{cos}\left({\phi }_{i}-{\phi }_{j}+{\phi }_{k}-{\phi }_{l}\right)+1\right]-\frac{{C}_{s}}{2}\sum_{i=1}^{N}\mathrm{cos}\left(2{\phi }_{i}\right)$$
	Dynamics: $$\frac{d{\phi }_{i}}{dt}=C\sum_{m=1}^{M}\left[\sum_{j=1}^{N}{c}_{mi}{c}_{mj}\mathrm{sin}\left({\phi }_{i}-{\phi }_{j}\right)+\sum_{\begin{array}{c}i\ne j\ne k\ne l\\ j<k<l\end{array}}^{N}{c}_{mi}{c}_{mj}{c}_{mk}{c}_{ml}\mathrm{sin}\left({\phi }_{i}-{\phi }_{j}+{\phi }_{k}-{\phi }_{l}\right)\right]-{C}_{s}\mathrm{sin}\left(2{\phi }_{i}\right)$$

Furthermore, using the dynamical system equation $\frac{d{\phi }_{i}}{dt}=-{\left({\nabla }_{\phi }E\right)}_{i}$, we can also show that for the energy functions described in Table 4, $\frac{dE}{dt}\le 0$ i.e., they are Lyapunov functions.

3 $$\frac{dE}{dt}=\sum_{i=1}^{N}\frac{\partial E}{\partial {\phi }_{i}}. \frac{d{\phi }_{i}}{dt}=\sum_{i=1}^{N}\left(-\frac{d{\phi }_{i}}{dt}\right) \frac{d{\phi }_{i}}{dt}=-\sum_{i=1}^{N}{\left(\frac{d{\phi }_{i}}{dt}\right)}^{2}$$

**Figure 1:**
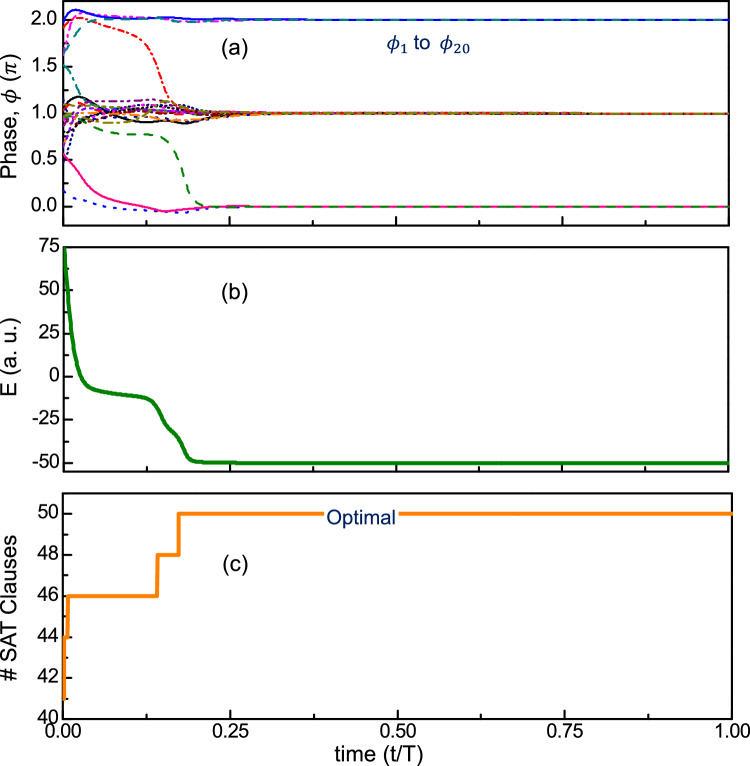
Evolution of (**a**) phases ($\phi$); (**b**) energy; (**c**) number of satisfied NAE-4-SAT clauses for an illustrative NAE-4-SAT problem (20 variables and 50 clauses) computed using the proposed dynamical system.

Figure 1 shows an illustrative example of the NAE-4-SAT problem computed using the proposed dynamical system. Details of the simulation used to simulate the illustrative NAE-4-SAT problem are described in Supplementary 4.

#### Max-K-Cut on a hypergraph

In the prior section, we exploited the binary nature of the Ising spins (along with higher order interactions among them). We now ‘extend’ the definition of the ‘spin’ in order to facilitate the design of computational models for an even broader spectrum of COPs that would benefit from the use of > 2 states for each node/spin. To facilitate this, we express the possible states of a spin as ${re}^{i{\theta }_{k}}$, where $r=1$, and ${\theta }_{k}=\frac{2\pi k}{K}$; $k=1, 2,\dots K-1$. When $K= 2$, the possible states are within {1, − 1}, which represents the traditional definition of an Ising spin. In contrast, when $K>2$, the ‘spin’ assumes $K$ configurations, represented as complex quantities (e.g., for $K=3$, the possible states are $1$, ${e}^{i\frac{2\pi (1)}{3}},{e}^{i\frac{2\pi (2)}{3}}$). While we have utilized this concept for solving combinatorial problems on *graphs* (i.e., problems with quadratic objective functions)^18^, here we explore this concept for hypergraphs (that entail higher order interactions) by considering the example of solving the Max-K-Cut of a hypergraph.

**Figure 2:**
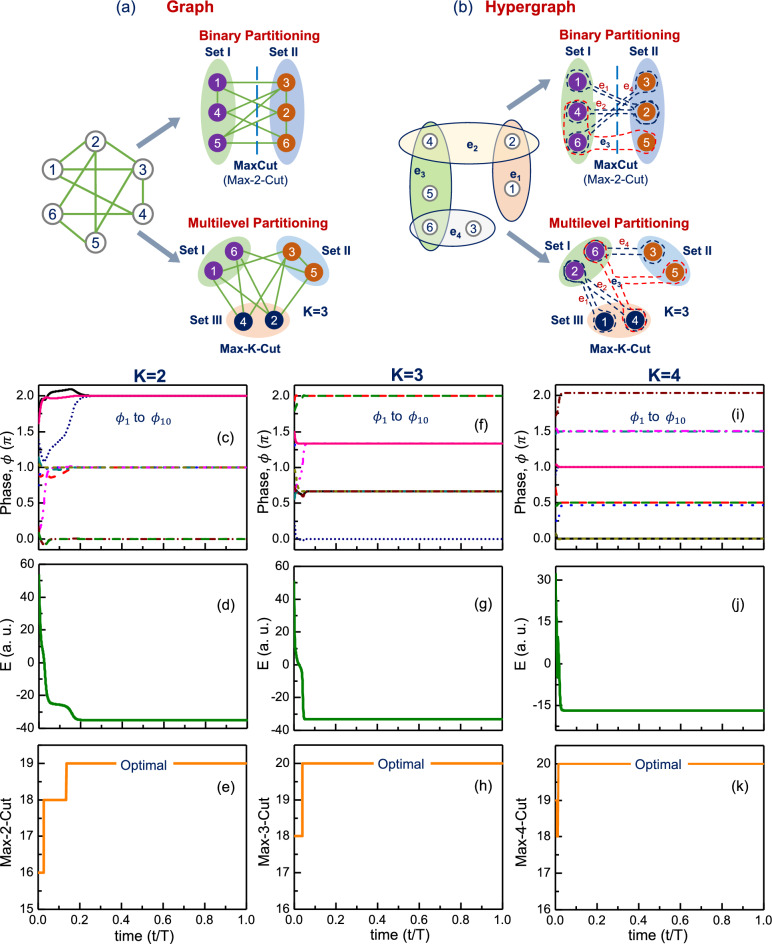
Illustrative example showing MaxCut and Max-K-Cut (K=3) on (**a**) graph; and (**b**) hypergraph. In case of hypergraph, ${e}_{i}$ denotes the *i*$\mathrm{th}$ hyperedge. Max-K-Cut (K = 2, 3, 4) solutions computed using the proposed dynamical system for an illustrative hypergraph. Evolution of phases ($\phi$), energy and the Max-K-Cut solution, respectively for (**c**–**e**) K = 2; (**f**–**h**) K = 3; (**i**–**k**) K = 4.

Computing the Max-K-Cut on a hypergraph is defined as the challenge of partitioning the nodes of a hypergraph into $K$ partitions in a manner that maximizes the number of hyperedges having nodes that lie in at least two sets created by the partitions^27^. The Max-K-Cut problem and its comparison with the archetypal MaxCut problem are illustrated in Fig. 2a,b for the case of a graph and a hypergraph, respectively.

To develop the objective function for the problem, each hyperedge of the graph can be expressed as ${h}_{m}=\prod_{i=1}^{N-1}\prod_{j=i+1}^{N}\left(1-{c}_{mi}{c}_{mj}\left(\frac{1-Re\left({s}_{i}{s}_{j}^{*}{e}^{\mathrm{i}f(\Delta {\theta }_{ij})}\right)}{2}\right)\right)$, where ${s}_{j}={1e}^{\mathrm{i}{\theta }_{j}}; {\theta }_{j}$ can assume any of the following values from $\frac{2\pi k}{K}$; $k=1, 2,\dots K-1$ enforced by the higher order harmonic injection. ${c}_{mj}=1$(0) if the ${j}{th}$ node belongs (does not belong) to the ${m}{th}$ hyperedge. We note that the $\tt  {^{\prime}}{i{^{\prime}}}$ represents the imaginary number $\sqrt{-1}$ whereas ${^{\prime}}i{^{\prime}}$ refers to the index.

4 $$f\left(\Delta {\theta }_{ij}\right)= \underset{\sigma \to 0}{\mathit{lim}}\sum_{k=1}^{K-1}\left[\left(\left(2k-1\right)\pi -\frac{2k\pi }{K}\right).{e}^{-\left(\frac{{\left(\Delta {\theta }_{ij}-\frac{2k\pi }{K}\right)}^{2}}{2{\sigma }^{2}}\right)}+\left(\frac{2k\pi }{K}-\left(2k-1\right)\pi \right).{e}^{-\left(\frac{{\left(\Delta {\theta }_{ij}+\frac{2k\pi }{K}\right)}^{2}}{2{\sigma }^{2}}\right)}\right]$$

$f(\Delta {\theta }_{ij})$ is designed such that $Re\left({s}_{i}{s}_{j}^{*}{e}^{\mathrm{i}f(\Delta {\theta }_{ij})}\right)=-1(1)$, if the nodes $i$ and $j$ are placed in different (same) sets, and essentially rewards (penalizes) the system in terms of energy, respectively. Additional details about the design and properties of $f(\Delta {\theta }_{ij})$ have been presented in our prior work^18^. Consequently, if the hyperedge satisfies the criterion for the Max-K-Cut i.e., that the nodes that are connected by it belong to at least two sets, the corresponding ${h}_{m}$ assumes a value of $0$, else ${h}_{m}=1$. Subsequently, the objective function for the problem, which entails maximizing the number of such hyperedges, can be expressed as minimizing $H$, where,

5 $$H=\sum_{m=1}^{M}{h}_{m}\equiv \sum_{m=1}^{M}\prod_{i=1}^{N-1}\prod_{j=i+1}^{N}\left(1-{c}_{mi}{c}_{mj}\left(\frac{1-Re\left({s}_{i}{s}_{j}^{*}{e}^{\mathrm{i}f(\Delta {\theta }_{ij})}\right)}{2}\right)\right)$$

As an example, considering a hypergraph where the maximum number of nodes connected by a hyperedge is 3, the objective function for the Max-K-Cut problem can be expressed as:

6 $$H=\sum_{\begin{array}{c}m=1,i\ne j\ne k\\ {c}_{mi},{c}_{mj},{c}_{mk}\ne 0\end{array}}^{M}\left(1-{c}_{mi}{c}_{mj}\left(\frac{1-Re\left({s}_{i}{s}_{j}^{*}{e}^{\mathrm{i}f\left(\Delta {\theta }_{ij}\right)}\right)}{2}\right)\right)\left(1-{c}_{mi}{c}_{mk}\left(\frac{1-Re\left({s}_{i}{s}_{k}^{*}{e}^{\mathrm{i}f\left(\Delta {\theta }_{ik}\right)}\right)}{2}\right)\right)\left(1-{c}_{mj}{c}_{mk}\left(\frac{1-Re\left({s}_{j}{s}_{k}^{*}{e}^{\mathrm{i}f(\Delta {\theta }_{jk})}\right)}{2}\right)\right)$$

where,

7 $$f\left(\Delta {\theta }_{ij}\right)= \underset{\sigma \to 0}{\mathit{lim}}\sum_{k=1}^{2}\left[\left(\left(2k-1\right)\pi -\frac{2k\pi }{3}\right).{e}^{-\left(\frac{{\left(\Delta {\theta }_{ij}-\frac{2k\pi }{3}\right)}^{2}}{2{\sigma }^{2}}\right)}+\left(\frac{2k\pi }{3}-\left(2k-1\right)\pi \right).{e}^{-\left(\frac{{\left(\Delta {\theta }_{ij}+\frac{2k\pi }{3}\right)}^{2}}{2{\sigma }^{2}}\right)}\right]$$

For a hypergraph with hyperedges having more than 3 nodes, the objective function entails the use of higher order interactions among the spins.

To formulate a dynamical system for minimizing the above objective function, we express $Re\left({s}_{i}{s}_{j}^{*}{e}^{\mathrm{i}f\left(\Delta {\theta }_{ij}\right)}\right)$ as $\mathrm{cos}\left(\Delta {\theta }_{ij}+f\left(\Delta {\theta }_{ij}\right)\right)$. Furthermore, we restrict the configuration space of $\theta$ to $\frac{2\pi k}{K}$ where $k=1, 2,\dots K-1$, by injecting the Kth harmonic (of sufficient strength) which lowers the energy at specific phase points, as described in prior work^18^. The resulting energy function can be described as,

8 $$E=A\sum_{m=1}^{M}\prod_{i=1}^{N-1}\prod_{j=i+1}^{N}\left(1-{c}_{mi}{c}_{mj}\left(\frac{1-\mathrm{cos}(\Delta {\phi }_{ij}+f(\Delta {\phi }_{ij}))}{2}\right)\right)-\frac{{A}_{s}}{K}\sum_{i=1}^{N}\mathrm{cos}(K{\phi }_{i})$$

We note that $\phi$ has been used to express the energy function for the dynamical system instead of $\theta$ which represents the configuration space of the ‘extended spin’. The corresponding dynamics for which the function in Eq. (8) is a Lyapunov function are given by:

9a $$\frac{d{\phi }_{i}}{dt}=-\frac{\partial E}{\partial {\phi }_{i}}$$

9b $$\frac{\mathrm{d}{\phi }_{i}}{\mathrm{dt}}=\frac{A}{2}\sum_{m=1}^{M}\sum_{j=1,j\ne i}^{N}\left[{c}_{mi}{c}_{mj}\mathrm{sin}\left(\Delta {\phi }_{ij}+f\left(\Delta {\phi }_{ij}\right)\right) \frac{{h}_{m}}{\left(1-{c}_{mi}{c}_{mj}\left(\frac{1-\mathrm{cos}\left(\Delta {\phi }_{ij}+f\left(\Delta {\phi }_{ij}\right)\right)}{2}\right)\right)}\right]-{A}_{s}\mathrm{sin}(K{\phi }_{i})$$

In the derivation of Eq. (9b), we exploit the fact that $\frac{\partial f\left(\Delta {\phi }_{ij}\right)}{\partial {\phi }_{i}}=0$^18^. Furthermore, using Eq. (9a), it can be shown that $\frac{dE}{dt}=-\sum_{i=1}^{N}{\left(\frac{d{\phi }_{i}}{dt}\right)}^{2} \le 0$ (similar to Eq. (3)).

We now evaluate our proposed model on a representative hypergraph. We consider a hypergraph where each hyperedge has 3 vertices. The corresponding dynamics for this case can then be written as,

10 $$\frac{\mathrm{d}{\phi }_{i}}{\mathrm{dt}}=\frac{A}{2}\sum_{m=1}^{M}\left[{c}_{mi}{c}_{mj}\mathrm{sin}\left(\Delta {\phi }_{ij}+f\left(\Delta {\phi }_{ij}\right)\right)\left(1-{c}_{mi}{c}_{mk}\left(\frac{1-\mathrm{cos}\left(\Delta {\phi }_{ik}+f\left(\Delta {\phi }_{ik}\right)\right)}{2}\right)\right)\left(1-{c}_{mj}{c}_{mk}\left(\frac{1-\mathrm{cos}\left(\Delta {\phi }_{jk}+f\left(\Delta {\phi }_{jk}\right)\right)}{2}\right)\right)+{c}_{mi}{c}_{mk}\mathrm{sin}(\Delta {\phi }_{ik}+f(\Delta {\phi }_{ik}))\left(1-{c}_{mi}{c}_{mj}\left(\frac{1-\mathrm{cos}(\Delta {\phi }_{ij}+f(\Delta {\phi }_{ij}))}{2}\right)\right)\left(1-{c}_{mj}{c}_{mk}\left(\frac{1-\mathrm{cos}(\Delta {\phi }_{jk}+f(\Delta {\phi }_{jk}))}{2}\right)\right)\right]-{A}_{s}\mathrm{sin}(K{\phi }_{i})$$

Figure 2c–k also shows the computed Max-K-Cut (for K = 2, 3, and 4) for a hypergraph instance (with 10 nodes, and 20 hyperedges). The illustrative problem has a maximum of 4 nodes per hyperedge. Details of the simulation used to simulate the illustrative Max-K-Cut problem are described in Supplementary 4.

## Conclusion

**Figure 3:**
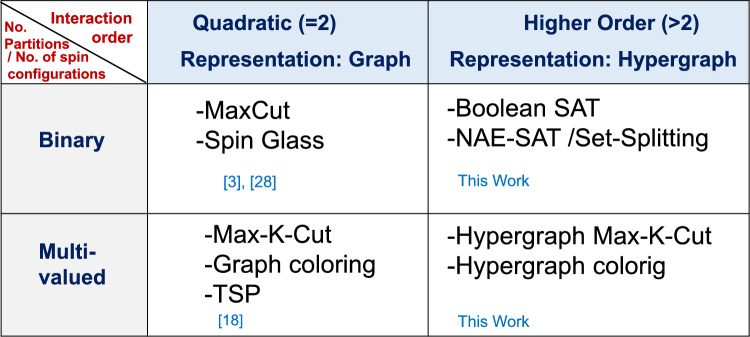
Proposed classification of COPs based on the number of states/configurations for the nodes, and the nature of interaction among them. Categorizing COPs helps develop a framework to formulate dynamical systems to solve the COPs. The present work enables the direct development of computational models for COPs that entail higher order interactions.

In this work, we develop computational models for Ising machines that consider higher order interactions (beyond quadratic/pairwise) among Ising spins. Our approach enables the direct formulation of analog computing models for many COPs that entail such interactions without the need for problem decomposition and reduction. Furthermore, using the combination of higher order interactions along with ‘expanding’ the number of ‘spin’ states to greater than 2, we can directly map and solve an even broader class of problems on hypergraphs. This has been summarized in Fig. 3. While the focus of the present work was to develop dynamical systems as higher order Ising machines, evaluating the scalability of this approach i.e., its ability to solve larger graphs will be crucial to its eventual success and utility. As the graph sizes increase, the role of local minima in the high dimensional phase space becomes increasingly important. The system dynamics may get trapped in such minima resulting in sub-optimal solutions. Furthermore, identifying the optimal range for the parameters ($C$ and ${C}_{s}$) in larger systems may also become more challenging. Eventually, these factors will also ascertain the performance benefits of this approach over traditional digital algorithms used to solve such problems. A systematic study to evaluate the scalability of this approach and its comparison with digital methods will be undertaken in the future. In the context of the broader effort focused on developing dynamical system-inspired models for solving hard COPs, this work expands on the potential of physics-inspired solvers to accelerate COPs.

## Supplementary Information

Supplementary Information.

## Data Availability

The data that support the findings of this study are available from the corresponding author upon reasonable request.
