# Supplementary material for: Designing Ising machines with higher order spin interactions and their application in solving combinatorial optimization
Source: Sci Rep. 2023 Jun 12;13:9558. doi: 10.1038/s41598-023-36531-4 (PMC10261086; doi:10.1038/s41598-023-36531-4)
Supplement: Supplementary file 1 — Supplementary Information. [file 41598_2023_36531_MOESM1_ESM.pdf]

Supplementary Material

Designing Ising Machines with Higher Order Spin Interactions and their Application in Solving Combinatorial Optimization

Mohammad Khairul Bashar, Nikhil Shukla

This supplement includes:

(1) Dynamical system for the NAE-5-SAT problem

(2) NAE-K-SAT results for an extended time

(3) Max-K-Cut results for an extended time

(4) Simulation method

1. Dynamical system for the NAE-5-SAT problem

Here, we develop the formulation of the objective function and the corresponding dynamical system for the NAE-5-SAT (K is an odd number) problem. An NAE-5-SAT clause can be represented as,

$$C = (x_i \oplus x_j) \vee (x_i \oplus x_k) \vee (x_i \oplus x_l) \vee (x_i \oplus x_m) \vee (x_j \oplus x_k) \vee (x_j \oplus x_l) \vee (x_j \oplus x_m) \vee (x_k \oplus x_l) \vee (x_k \oplus x_m) \vee (x_l \oplus x_m) \quad \text{(S1)}$$

In terms of Ising spins, the complement of C can be written as,

$$\left(\frac{1+s_is_j}{2}\right)\left(\frac{1+s_is_k}{2}\right)\left(\frac{1+s_is_l}{2}\right)\left(\frac{1+s_is_m}{2}\right)\left(\frac{1+s_js_k}{2}\right)\left(\frac{1+s_js_l}{2}\right)\left(\frac{1+s_js_m}{2}\right)$$

$$\left(\frac{1+s_ks_l}{2}\right)\left(\frac{1+s_ks_m}{2}\right)\left(\frac{1+s_ls_m}{2}\right)$$

$$= \frac{1}{2^4}\left(1 + s_is_j + s_is_k + s_is_l + s_is_m + s_js_k + s_js_l + s_js_m + s_ks_l + s_ks_m \right. \quad \text{(S2)}$$

$$\left. + s_ls_m + s_is_js_ks_l + s_is_js_ks_m + s_is_js_ls_m + s_is_ks_ls_m + s_js_ks_ls_m\right)$$

Thus, the objective function for an NAE-5-SAT problem with M clauses can be written as,

$$H = -\sum_{m=1}^{M}\left(\sum_{\substack{i,j \\ i<j}}^{N}\left(-c_{mi}c_{mj}s_is_j\right) + \sum_{\substack{i,j,k,l \\ i<j<k<l}}^{N}\left(-c_{mi}c_{mj}c_{mk}c_{ml}s_is_js_ks_l\right)\right) \quad \text{(S3)}$$

Where, $c_{mi} = -1$ $(+1)$ if the ith variable appears in inverted (normal) form in the mth clause; $c_{mi} = 0$ if the ith variable is absent in the mth clause.

Using the approach described in the main text, the corresponding Lyapunov function and the system dynamics can be formulated as,

Energy:

$$E = C\sum_{m=1}^{M}\left[\sum_{i,j,i<j}^{N} c_{mi}c_{mj}\cos\left(\phi_i - \phi_j\right) + \sum_{\substack{i,j,k,l\\ i<j<k<l}}^{N} c_{mi}c_{mj}c_{mk}c_{ml}\cos\left(\phi_i - \phi_j + \phi_k - \phi_l\right) + 1\right] - \frac{C_s}{2}\sum_{i=1}^{N}\cos(2\phi_i) \tag{S4}$$

Dynamics:

$$\frac{d\phi_i}{dt} = C\sum_{m=1}^{M}\left[\sum_{j=1}^{N} c_{mi}c_{mj}\sin\left(\phi_i - \phi_j\right) + \sum_{\substack{i\neq j\neq k\neq l\\ j<k<l}}^{N} c_{mi}c_{mj}c_{mk}c_{ml}\sin\left(\phi_i - \phi_j + \phi_k - \phi_l\right)\right] - C_s\sin(2\phi_i) \tag{S5}$$

Equations (S3), (S4), and (S5) are also shown in Table 4 in the main text.

2. NAE-4-SAT results for an extended time

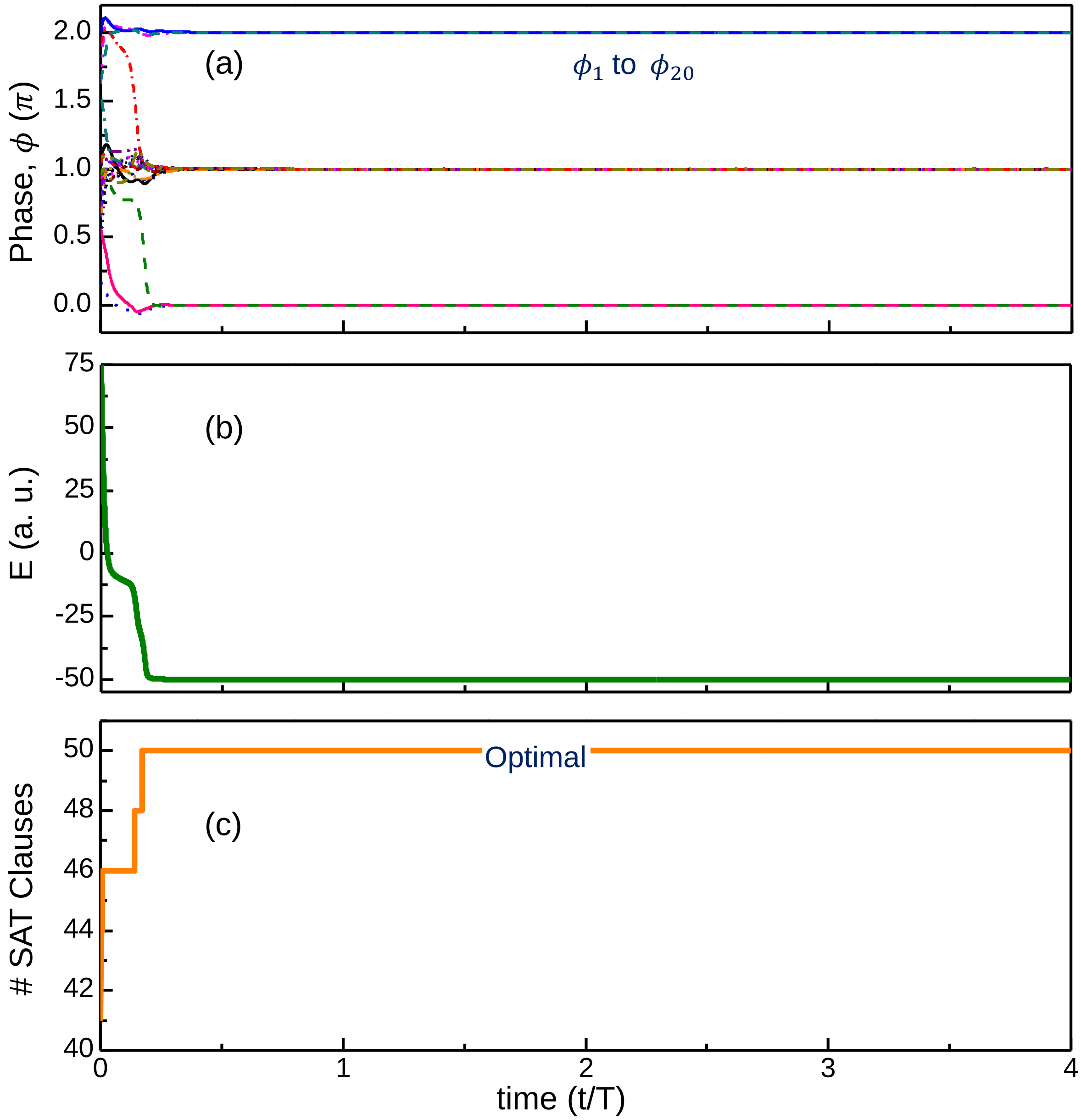

Figure S1. Evolution of (a) phases (ϕ); (b) energy; (c) number of satisfied NAE-4-SAT clauses for an illustrative NAE-4-SAT problem (20 variables and 50 clauses) computed using the proposed dynamical system.

Figure S1 shows the evolution of the phases, energy, and number of satisfied NAE-4-SAT clauses in Fig. 1 of the main manuscript over a longer time period (4 cycles).

3. Max-K-Cut results for an extended time

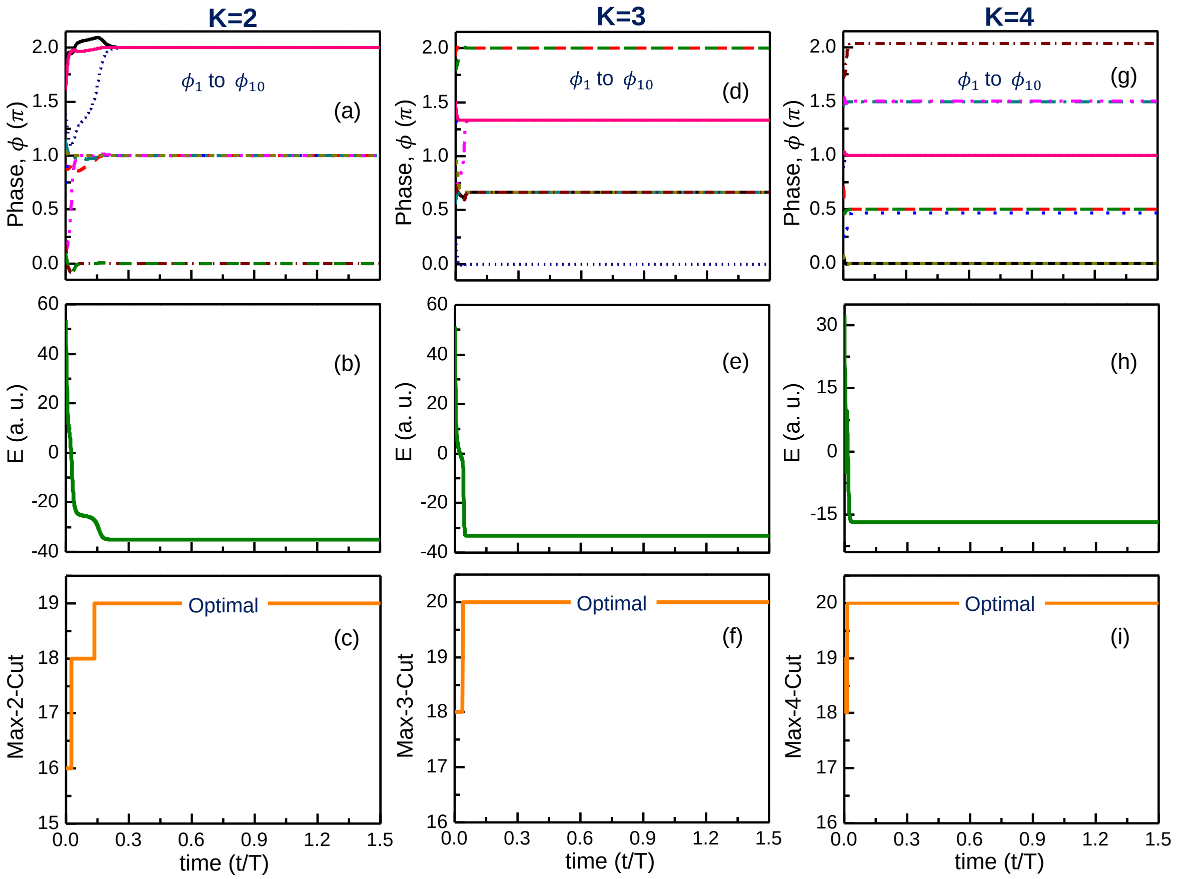

Figure S2. Max-K-Cut (K=2,3,4) solutions computed using the proposed dynamical system for an illustrative hypergraph. Evolution of phases (ϕ), energy and the Max-K-Cut solution, respectively for (a-c) K=2; (d-f) K=3; (g-i) K=4.

Figure S2 shows the evolution of the phases, energy, and Max-K-Cut solution (K=2, 3, and 4) in Fig. 2 of the main manuscript over a longer time period (1.5 cycles).

4. Simulation method

Here, we describe the simulation approach used to simulate the NAE-4-SAT problem (Fig. 1, main text) and the hypergraph Max-K-Cut problem (Fig. 2, main text). We solve the dynamics using a stochastic differential equation (SDE) solver implemented in MATLAB; details of its implementation have been described in our previous work [S1]. The SDE solver incorporates noise that helps escape local minima in the phase space.

Values of C and C_s used in the simulation of the NAE-4-SAT are:

Problem Solved	$\boldsymbol{C}$	$\boldsymbol{C_s}$
NAE-4-SAT	$\frac{10}{8}$	5

Values of A and A_s used in the simulation of the Max-K-Cut are:

Problem Solved	$\boldsymbol{A}$	$\boldsymbol{A_s}$
Hypergraph Max-2-Cut	15	10
Hypergraph Max-3-Cut	15	10
Hypergraph Max-4-Cut	10	10

References

S1. Bashar, M.K., Lin, Z. and Shukla, N. Oscillator-Inspired Dynamical Systems to Solve Boolean Satisfiability. IEEE Journal on Exploratory Solid-State Computational Devices and Circuits, Early Access (2023). DOI: https://doi.org/10.1109/JXCDC.2023.3241045
